# *Acidobacteria* appear to dominate the microbiome of two sympatric Caribbean Sponges and one Zoanthid

**DOI:** 10.1186/0717-6287-47-67

**Published:** 2014-12-10

**Authors:** Aileen O’Connor-Sánchez, Adán J Rivera-Domínguez, César De los Santos-Briones, Lluvia K López-Aguiar, Yuri J Peña-Ramírez, Alejandra Prieto-Davo

**Affiliations:** Unidad de Biotecnología. Centro de Investigación Científica de Yucatán A.C, Calle 43 # 130, Chuburná de Hidalgo, 97200 Mérida, Yucatán México; Facultad de Química - Unidad Académica Sisal, Universidad Nacional Autónoma de México, Puerto de abrigo s/n, Municipio de Hunucmá, 97356 Sisal, Yucatán, México; Departamento de Ciencias de la Sustentabilidad, El Colegio de la Frontera Sur - Unidad Campeche, Campeche, México

**Keywords:** Marine-invertebrate microbiome, 16S rRNA pyrotags, Mexican Caribbean, Sponge-microbiome, Zoanthid-microbiome, Marine sediments-microbiome

## Abstract

**Background:**

Marine invertebrate-associated microbial communities are interesting examples of complex symbiotic systems and are a potential source of biotechnological products.

**Results:**

In this work, pyrosequencing-based assessment from bacterial community structures of sediments, two sponges, and one zoanthid collected in the Mexican Caribbean was performed. The results suggest that the bacterial diversity at the species level is higher in the sediments than in the animal samples. Analysis of bacterial communities’ structure showed that about two thirds of the bacterial diversity in all the samples belongs to the phyla *Acidobacteria* and *Proteobacteria*. The genus *Acidobacterium* appears to dominate the bacterial community in all the samples, reaching almost 80% in the sponge Hyrtios.

**Conclusions:**

Our evidence suggests that the sympatric location of these benthonic species may lead to common bacterial structure features among their bacterial communities. The results may serve as a first insight to formulate hypotheses that lead to more extensive studies of sessile marine organisms’ microbiomes from the Mexican Caribbean.

**Electronic supplementary material:**

The online version of this article (doi:10.1186/0717-6287-47-67) contains supplementary material, which is available to authorized users.

## Background

In recent years, the studies on sponge-associated microbial communities have had an increased attention of the scientific community. The two most important reasons for this are: (I) the secondary metabolites produced by the sponge microbiome have shown very promising pharmaceutical and biotechnological activities (e.g. as antibacterial, anthelmintic, anti-inflammatory, or antitumor agents, or neurosuppressors) [1]. (II) The sponge-microbe interactions are interesting examples of complex symbiotic systems [2].

Sponges are the simplest multicellular animals and the most ancient metazoans [3]. The microorganisms they contain are integral components of their body and can account for up to 40% of their volume [4]. As filter feeders, sponges are capable of turning over many thousands of liters of water per day; prokaryotes, as well as nano- and pico-eukaryotes, are the most important components of the sponge diet, but they also have other roles, as pathogens or symbionts, or in stabilizing sponge skeleton or processing metabolic waste [4, 5]. Sponge-microbe interactions are complex, and some evidence supports high host specificity among many of these associations [6], suggesting that each sponge exerts a selective pressure determining the structure of its bacterial community. Furthermore, possibly the sponge together with its specific microbial community prevents the establishment of other microorganisms in the sponge body. Nevertheless, some studies show that sponge-associated bacterial communities may suffer modifications according to environmental conditions [7], suggesting that although the relationship among bacteria and sponge may be specific, it may also show aspects of dynamism.

Numerous studies have reported the existence of sponge-specific 16S ribosomal RNA gene sequence clusters, describing bacteria found in sponges but very rare in surrounding environments, like sediments or water [8]. The diversity and specificity of microbial communities in marine organisms is a key aspect of the ecology and evolutionary relationships between both the eukaryotic hosts and their associated prokaryotes. To date, 32 major bacterial phyla and several possibly novel sponge-associated bacterial communities have been identified [9]. Nevertheless, most studies have focused on sponges of high latitudes and very little is known about the structure of microbial communities associated with sponges of tropical seas.

The recent advent of massively parallel sequencing technologies has revolutionized microbial diversity and ecology studies. The gene encoding the small-subunit of the rRNA serves as a prominent tool for the phylogenetic analysis and classification of bacteria, owing to its high degree of conservation and its fundamental function in living organisms [10]. Pyrosequencing of this gene has proved to be a cost-effective method for the characterization of bacterial communities and, although it may be subject to a moderate bias [11], it is widely used to get a cultivation-independent general view about the phylogenetic profile of bacterial communities (e.g. [8]).

The aim of the present work was to analyze by 16S tag-pyrosequencing assessment, the bacterial community structures of three sympatric marine organisms (two sponges and one zoanthid) and the sediments beneath them in a location of the Caribbean Sea.

## Results and discussion

A total of 26,959 rRNA quality sequences with an average read length of 466 bp were generated through the tag-pyrosequencing. At phylum level, all the samples had a similar number of OTUs, ranging from 44 to 49, with exception of the sponge *Hyrtios*, which had only 28. Nevertheless, at species level, the sediments showed a larger number of OTUs (441) than did the animal samples. The zoanthid *Palythoa* had 385 OTUs, while the sponges *Aiolochroia* and *Hyrtios* had 279 and 127 respectively (Table 1).

**Table 1:** **General analysis of the pyrosequencing-derived datasets**

			***20% Dissimilarity***				***3% Dissimilarity***			
***Sample***	***# Reads***	***Av seq len***	***# OTUs***	***Chao I***	***Shannon (H’)***	***Evenness***	***# OTUs***	***Chao I***	***Shannon (H’)***	***Evenness***
Sediments	2,348	413	45	45	2.95	0.77	441	460	5.52	0.90
*Palythoa*	12,073	478	44	44	2.72	0.72	385	411	3.98	0.66
*Aiolochroia*	6,439	464	49	49	2.74	0.70	279	308	3.95	0.70
*Hyritios*	6,099	466	28	28	1.30	0.39	127	164	1.67	0.34

The number of OTUs, Shannon diversity, Chao I and evenness were analyzed at 20% (the phylum level) and 3% (the species level) sequence dissimilarity for each sample.

Chao 1 richness estimator suggests that most of the estimated diversity contained within these communities was captured by our sequencing efforts; even in the case of the sediments, where less reads were generated. Actually, at phylum level, the Chao 1 index coincides with the number of detected OTUs for all the samples. At species level, the sediments had the highest diversity predicted by Chao1 (460), while the sponge *Hyrtios* showed the lowest (164).

The Shannon diversity index values (*H’*), both at the phylum and species levels, suggest that the sediments held a higher bacterial diversity than the marine organisms living right above them; among which the zoanthid *Palythoa* had about the same bacterial diversity at species level than the sponge *Aiolochroia* and a more diverse bacterial community than the sponge *Hyrtios*. The sediments also presented higher evenness values than the animal samples; meaning that they had not only a higher number of taxa, but that their relative abundances were more homogeneous; thus making them the most diverse sample in this study. The higher bacterial diversity observed in the sediments is not surprising and has been described before [12]; it is also most likely the result of the sediments being subjected to more variable environmental conditions than the inside of the animal tissues, where highly selective conditions should prevail; furthermore, marine sediments may represent an environment with a higher number of microniches than those in animal tissue. All the observed values in our sponges are typically found in sponge microbiomes (e.g. [13]), which supports the former hypothesis.

**Figure 1:**
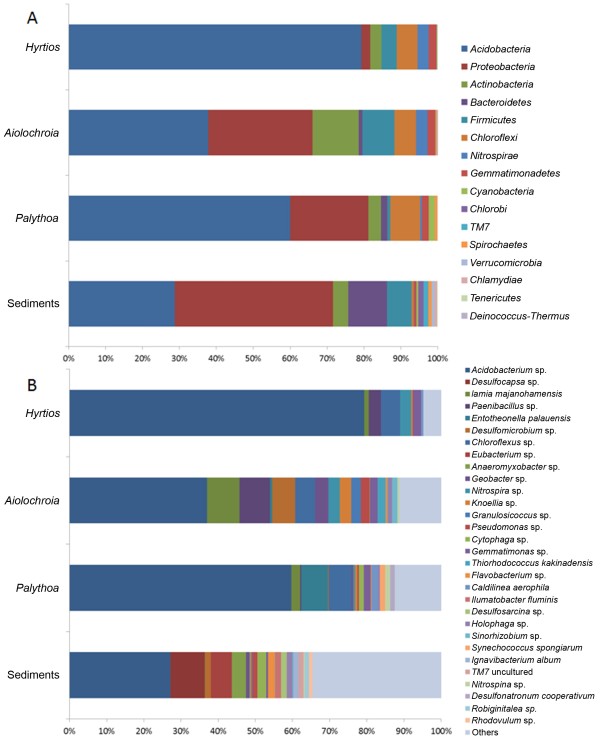
**Bacterial distribution among the different marine samples.** Percentage of different bacteria at **A)** the phylum, and **B)** the species level (20% and 3%, respectively) present in the three marine organisms and sediment sample from the Mexican Caribbean. *Acidobacteria* dominate all three marine organisms while *Proteobacteria* dominate the marine sediment sample.

All the generated reads were classified as belonging to the domain Bacteria. The reads from all the four samples together represented a total of 16 phyla (Figure 1A), out of which 12 had a relative abundance of >1% in at least one sample. The phyla *Chlorobi*, *TM7* and *Tenericutes* were only observed in the sediments sample, while the phylum *Deinococcus-Thermus* was only found in the zoanthid *Palythoa* and in the sponge *Aiolochroia*, and the only sample where the phylum *Chlamydiae* was not detected was the sponge *Hyrtios*. All the other phyla were present in the four samples. The two most dominant phyla in all the samples were *Acidobacteria* and *Proteobacteria*. Together, both accounted for more than two thirds of the total phyla present in each sample (Figure 1A). The third most abundant phylum within the sediments was *Bacterioidetes*, with 10%, while in the animal samples it showed considerably lower abundances, ranging from 0.06% to 1.77%. In the zoanthid and in the sponge *Hyrtios*, the third most abundant phylum was *Chloroflexi* (8% and 6% respectively), while in *Aiolochroia*, this rank was taken by *Actinobacteria* (12%)*.* All the phyla found in the animal samples have been previously reported as part of sponge microbiomes [8] while some phyla commonly found in sponge microbiomes, like *Planctomycetes* and the candidate phylum *Poribacteria*, were not observed when using the RDP pipeline presented in this study.

The most outstanding difference in the bacterial community structure between the sediments and the animal samples was that while the sediments were dominated by *Proteobacteria*, the animal samples were strongly dominated by *Acidobacteria*. To our knowledge, this is the first report of *Acidobacteria* reaching abundances up to 79% in a sponge microbiome, where the dominant phyla that have been reported are either *Proteobacteria* (e.g. [9, 14]) or *Chloroflexi*[2, 13].

Another surprising finding in this work was that at 3% dissimilarity, *Acidobacteria* grouped in only one cluster (Figure 1B). However, when closely analyzing this data using the program Qiime [15], which uses the “green-genes” database (http://greengenes.lbl.gov/cgi-bin/nph-index.cgi), it was seen that this cluster contained 13 OTUs represented by uncultivated clones, and *Candidatus Solibacter* (Additional file 1: Figure S1). Nevertheless it is clear that this group contains only bacteria of the phylum *Acidobacterium*. In contrast, the phylum *Proteobacteria* split into several species, supporting previous observation about this phylum being one of the most diverse in sponges [6]. Due to the species partitioning observed in *Proteobacteria*, the genus *Acidobacterium* (phylum *Acidobacteria*, class *Acidobacteria*) was the most abundant in all the samples we tested, including the sediments (Figure 1B).

To our knowledge, there are only two previous studies on the bacterial community structures in sponges of the genus *Hyrtios*. (i) Schmitt et al. [6] studied the microbiome of three *Hyrtios* species: *Hyrtios* sp. collected in the Great Barrier Reef (Australia), *Hyrtios altum* collected in Guam (Northern Pacific Ocean), and *Hyrtios erectus* collected in the Red Sea. Although their analyses were not reported at the species level, the study does not reveal a clear correlation between microbial community structure and host phylogeny and suggests that geographic location may have a greater effect on microbial community structure than does the identity of the host sponge. (ii) Recently, Jeong et al. [13] studied the bacterial community structure present in two samples of *Hyrtios erectus* collected in Micronesia, where *Acidobacteria* represented only 10-20% of the total bacterial abundance in the sponges; far from the 79% observed in the *Hyrtios* sample in the present study. Our data strengthens the hypothesis that, although sponge-associated microbial communities may have some degree of host specificity, environmental conditions may exert a stronger influence on bacterial selection, thus the bacterial community structures of sponge microbiomes can have variations according to the geographic location where the organism is settled.

Meanwhile, only one study has been done on the microbiome of a zoanthid. Sun et al. [16] analyzed *Palythoa australiae* samples collected in the South China Sea and found that *Proteobacteria* was the most abundant phylum in that organism with a relative abundance of 58.6 %, followed by *Chloroflexi* (12%) and *Actinobacteria* (10%). *Acidobacteria* accounted for only 6% of the phyla they detected; in contrast with the 60% that we found in our zoanthid sample. As for sediment samples, our results agree with previous studies on the community structure in sea sediments (e.g. [17]), where *Proteobacteria* has been reported as the dominant phylum.

Our study suggests that sympatric samples may share some patterns on bacterial community structure and that the geographic location may have an influence as strong –or even stronger- than the phylogenetic relationship among their host organisms.

Members of the phylum *Acidobacteria* have been observed in many different habitats. Their phylogenetic diversity, ubiquity and abundance suggest that they have an important ecological role and an extensive metabolic versatility [18]. However, the genetic and physiological information regarding *Acidobacteria* is very scarce, as the majority of its members have not been cultivated and they have only been identified by their 16S rDNA sequences. Currently, only 17 genome sequences from this phylum are publicly available [19].

## Conclusions

Our results suggest that the environmental conditions in the sampled location of the Caribbean Sea, together with the conditions in the tissue of the studied samples, exerted a selective pressure favoring bacteria from the genus *Acidobacterium*. There is a possibility that the host, together with its associated *Acidobacteria* creates conditions that strongly inhibit the proliferation of other taxa (e.g. by producing secondary metabolites). Further work would be necessary to test these hypotheses, and support the study of these marine invertebrate genera as a source of novel marine natural products. Our study is a first insight into the microbiome of sessile marine invertebrates from the Mexican Caribbean using cultivation independent methods, and it may serve to formulate future hypotheses that contribute to a better scientific understanding of the marine organisms-microbiome interactions and their biotechnological potential.

## Methods

Two sponges identified as *Aiolochroia* sp. (phylum *Porifera*, class *Demospongias*, order *Verongida*) and *Hyrtios* sp. (phylum *Porifera*, class *Demospongias*, order *Dictyoceratida*), one zoanthid identified as *Palythoa* sp. (phylum *Cnidaria*, class *Anthozoa*, order *Zoanthidea*), and a sediment sample, were collected by scuba diving at a depth of about 4–10 m in the Mexican area of the Caribbean Sea, in the reefs surrounding the lagoon of Banco Cinchorro (18° 23.66 N; 87° 24.447 W ), in July 2012. The three sampled organisms and the sediments were collected very near to each other, within a radius of about 1 m. The four samples were collected aseptically, enclosed in sterile bags, frozen at -20°C, and immediately transported to the laboratory.

Animal samples were then thawed and cut into small pieces and homogenized in a sterilized mortar. Metagenomic DNA was extracted according to the method described by Taylor et al. [20]. The DNA from the sediment sample was extracted following the method reported by Rojas-Herrera et al. [21].

Purified metagenomic DNA was submitted to the Research and Testing Laboratory (RTL) (Lubbock, TX, USA) for tag-pyrosequencing. Bacterial tag-encoded FLX amplicon pyrosequencing (bTEFAP) was performed as described previously using bacterial universal primers Gray28F (5’-TTTGATCNTGGCTCAG-3’) and Gray519R (5’-GTNTTACNGCGGCKGCTG-3’) primers [22]. Sequencing was based on RTL protocols (http://www.researchandtesting.com).

Following sequencing, all failed sequence reads, low quality sequence ends and tags and primers were removed and sequence collections depleted of any non-bacterial ribosome sequences and chimeras using the B2C2 software [23], as described previously [24]. To determine the identity of bacteria in the remaining sequences, these were denoised, assembled into clusters and queried using a distributed BLASTn.NET algorithm against a database of high-quality 16S bacterial sequences derived from NCBI. Database sequences were characterized as high-quality based upon criteria similar to those utilized by RDP [25]. Using a .NET and C# analysis pipeline, the resulting BLASTn outputs were compiled and validated using taxonomic distance methods and data reduction analysis was performed as described previously [24]. Based upon the above BLASTn-derived sequence identity (percent of total-length query sequence which aligns with a given database sequence) and validated using taxonomic distance methods, the bacterial sequences were classified at the appropriate taxonomic levels based upon the following criteria: sequences with identity scores (relative to known or well characterized 16S sequences) greater than 97% identity (<3% divergence) were resolved at the species level, and 77% to 80% at the phylum level. Sequencing reads were aligned and clustered following the Ribosomal Database Project (RDP-Release 10) pyrosequencing pipeline (http://pyro.cme.msu.edu/). Shannon, Chao 1, and evenness indices were obtained using the RDP tools.

All the 16S rRNA gene sequences were deposited in the NCBI Sequence Read Archive (SRA) under the Bioproject PRJNA256178 (http://www.ncbi.nlm.nih.gov/biosample?LinkName=bioproject_biosample_all_&from_uid%20=%20256178).

All the experimental research that is reported in the manuscript has been performed with the approval of an appropriate ethics committee. There was not experimental research neither on humans nor on animals.

## Electronic supplementary material

Additional file 1: Operational taxonomic units (OTUs) at 97% 16S rRNA sequence similarity belonging to the phylum Acidobacteria that were observed in the three sympatric marine organisms studied. (PDF 90 KB)
