# Supplementary figures and images for: Acidobacteria appear to dominate the microbiome of two sympatric Caribbean Sponges and one Zoanthid
Source: Biol Res. 2014 Dec 10;47(1):67. doi: 10.1186/0717-6287-47-67 (PMC4335776; doi:10.1186/0717-6287-47-67)

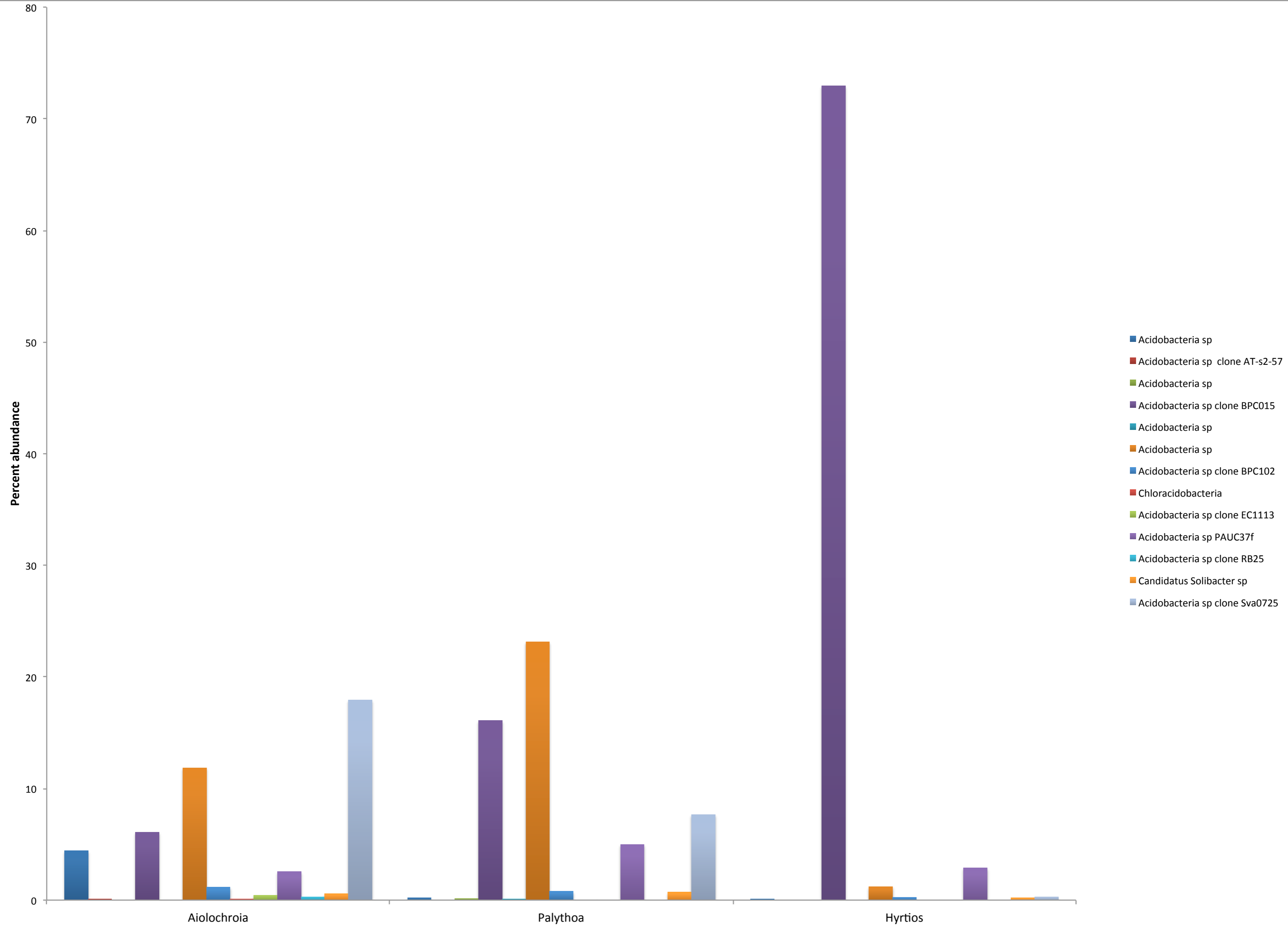

Supplement: Supplementary file 1 — Additional file 1: Operational taxonomic units (OTUs) at 97% 16S rRNA sequence similarity belonging to the phylum Acidobacteria that were observed in the three sympatric marine organisms studied. (PDF 90 KB) [file 40659_2014_77_MOESM1_ESM.pdf]
